# Docetaxel-Loaded Methoxy poly(ethylene glycol)-poly (L-lactic Acid) Nanoparticles for Breast Cancer: Synthesis, Characterization, Method Validation, and Cytotoxicity

**DOI:** 10.3390/ph16111600

**Published:** 2023-11-13

**Authors:** Shumaila Miraj, Hamid Saeed, Mehwish Iqtedar, Norah A. Albekairi, Nadeem Ahmed, Muhammad Zeeshan Danish, Muhammad Islam, Muhammad Fawad Rasool, Kashif Mairaj Deen, Hassaan Anwer Rathore

**Affiliations:** 1 Department of Pharmaceutics, College of Pharmacy, University of the Punjab, Lahore 54000, Pakistan; shumaila.miraj@yahoo.com (S.M.); zeeshan.pharmacy@pu.edu.pk (M.Z.D.); islam.pharmacy@pu.edu.pk (M.I.); 2 Department of Biotechnology, Lahore College for Women University, Jail Road, Lahore 54000, Pakistan; miqtedar@gmail.com; 3 Department of Pharmacology and Toxicology, College of Pharmacy, King Saud University, P.O. Box 2455, Riyadh 11451, Saudi Arabia; 4 Center of Excellence in Molecular Biology, University of the Punjab, Lahore 54590, Pakistan; nadeem.cemb@pu.edu.pk; 5 Faculty of Pharmacy, Bhauddin Zakariya University, Multan 59071, Pakistan; fawadrasool@bzu.edu.pk; 6 Department of Materials Engineering, The University of British Columbia, Vancouver, BC V6T 1Z4, Canada; kashifmairaj.deen@ubc.ca; 7 College of Pharmacy, Qatar University, Doha 2713, Qatar; hrathore@qu.edu.qa

**Keywords:** docetaxel, mPEG-PLA, nanoparticles, breast cancer, validation method

## Abstract

This study aimed to synthesize and characterize DTX-mPEG-PLA-NPs along with the development and validation of a simple, accurate, and reproducible method for the determination and quantification of DTX in mPEG-PLA-NPs. The prepared NPs were characterized using AFM, DLS, zetasizer, and drug release kinetic profiling. The RP-HPLC assay was developed for DTX detection. The cytotoxicity and anti-clonogenic effects were estimated using MTT and clonogenic assays, respectively, using both MCF-7 and MDA-MB-231 cell lines in a 2D and 3D culture system. The developed method showed a linear response, high precision, accuracy, RSD values of ≤2%, and a tailing factor ≤2, per ICH guidelines. The DTX-mPEG-PLA-NPs exhibited an average particle size of 264.3 nm with an encapsulation efficiency of 62.22%. The in vitro drug kinetic profile, as per the Krosmeyers–Peppas model, demonstrated Fickian diffusion, with initial biphasic release and a multistep sustained release over 190 h. The MTT assay revealed improved in vitro cytotoxicity against MCF-7 and MDA-MB-231 in the 2D cultures and MCF-7 3D mammosphere cultures. Significant inhibitions of the clonogenic potential of MDA-MB-231 were observed for all concentrations of DTX-mPEG-PLA-NPs. Our results highlight the feasibility of detecting DTX via the robust RP-HPLC method and using DTX-mPEG-PLA-NPs as a perceptible and biocompatible delivery vehicle with greater cytotoxic and anti-clonogenic potential, supporting improved outcomes in BC.

## 1. Introduction

The incidence of breast cancer (BC) is increasing at an alarming rate, with more than 50% of cases reported in the Punjab province of Pakistan only, and almost 40,000 women die per year from this disease, the highest among Asian countries [1]. Despite advancements in the field of BC therapy, approximately 40% of BC patients suffered disease recurrence and poor survival. Thus, BC remains the most common cause of death in women worldwide [2]. Among other variables, drug resistance is clinically the most critical factor contributing to BC recurrence, which can be managed by identifying more specific and targeted therapeutic agents [3]. In this context, nanoscale delivery vehicles have been used as versatile carriers for the transport of already-tested anticancer drugs to the target tumor sites because of their unique properties [4,5]. Nanoparticles are therapeutically efficient as they demonstrate fewer side effects and depict an advantage over conventional treatment strategies [6,7]. In addition, nanoparticles, with proven in vivo stability, have been shown to improve the drug solubility of hydrophobic drugs and are efficient vehicles for achieving extended steady-state drug levels at the tumor site, being attributed to improving pharmacokinetic profiles with reduced multidrug resistance [8,9].

Recently, polymeric nanoparticles based on amphiphilic block polymers have represented an attractive class of nanocarriers that can build a strong core–shell structure for loading both aqueous and lipophilic chemotherapeutic drugs and stabilize the colloids in conditions where the self-assembly properties are needed [10,11,12]. Therefore, these polymers are of great interest because of their distinct chemical structure and properties. Furthermore, they have much less nano–bio interaction with the reticuloendothelial system-associated clearance mechanism and protein absorption, which certainly aids in prolonging the retention time and the circulation of these polymeric nanoparticles in the bloodstream [13]. Additionally, nanoparticles have been shown to preferentially improve therapeutic efficacy by achieving extended steady-state drug levels at the tumor site, which have been attributed to improved pharmacokinetic profiles with reduced toxic effects and multidrug resistance [8,9].

Methoxy poly (ethylene glycol)-poly (L-lactic acid) (mPEG-PLA) is an example of an amphiphilic block copolymer that has been used extensively in recent years for the delivery of anticancer drugs because of its biodegradable nature and low clearance rate [14]. Compared with homopolymers, this co-polymer is a more suitable candidate for drug loading due to its high biocompatibility, high encapsulation efficiency, and drug release pattern with a low initial burst release [15]. Additionally, the presence of hydrophilic poly (ethylene glycol) (PEG) chains makes these polymeric nanoparticles more resistant to opsonization and phagocytosis, and they can be modified to escape the mononuclear phagocytic system (MPS) upon intravenous administration [16]. Interestingly, a PEG-PLA-based micellar formulation of paclitaxel has been registered in Korea with the trading name of Genexol^®^-PM, and it is being prescribed for BC, ovarian cancer, and lung cancer treatment [17,18]. This polymer has been used to co-deliver verapamil and doxorubicin in ovarian cancer, and it has demonstrated an improved pharmacokinetic profile, better anticancer response, less systemic toxicity, and drug resistance [19].

Docetaxel (DTX) is an FDA-approved semisynthetic lipophilic anticancer drug of the Taxoid family derived from *Texus baccata* [20], with proven efficacy against solid tumors such as breast, lung, ovarian, prostate, and gastric adenocarcinoma and tumors [21,22,23,24]. Despite its preferential usage in clinics due to its high binding affinity for microtubules compared with paclitaxel [25], the fullest clinical potential of docetaxel is marred by its low water solubility, high lipophilicity, low bioavailability, allergic reactions, and systemic toxicity [26]. However, these limitations can be overcome by designing nanoformulations such as polymeric, magnetic, inorganic, and liposome nanocarrier systems [23,27,28]. Aside from that, docetaxel has been formulated as PEGylated liposomes, demonstrating reduced uptake via the mononuclear phagocytic system (MPS) and a prolonged retention time when studied in mice [29]. Nonetheless, to fully characterize and ensure the quality benefits of polymeric nanoparticles, a suitable validation method and a vital analytical tool that ensures the accuracy and specificity of the analytical procedures are required to determine the detection and quantitation limit for the estimation of drug components [30]. Therefore, it can be inferred that the nanomedicine application is promising for executing site-specific treatment compared with free drugs, which has some associated drawbacks for drug stability, pharmacokinetics, targeting, safety, and multifunctionality [31].

The docetaxel polymeric micellar nanoparticles have been developed with the name CriPec^®^, and its assay method has been validated via liquid chromatography–tandem mass spectrometry on human plasma and tissue samples after treatment with this dosage form [32]. Yet, there is scant literature evidence on the use of amphiphilic polymer for DTX loading and DTX validation in the polymeric formulation. Thus, using amphiphilic polymer, we aimed to encapsulate DTX into the mPEG-PLA polymer, followed by its characterization employing avante garde modalities. So far, no DTX validation method has been developed to determine DTX in mPEG-PLA polymer. Therefore, we further aimed to develop a simple and robust DTX validation method using ultra high-performance liquid chromatography with a photodiode array detector followed by their cytotoxic and anti-clonogenic potential against BC cell lines.

## 2. Results

### 2.1. Linearity and Range

The developed method showed a linear response in the range of 0.64–120 µg/mL for the analyte with a correlation coefficient of 0.997. The representative regression equation for the straight line was y = 12,202,476x − 2188.3, where 2188.3 is the y-intercept, *x* indicates the concentration, and the slope is 12,202,476 (Figure S1A).

### 2.2. Limit of Detection and Limit of Quantification

The limit of detection (LOD) and limit of quantification (LOQ) were obtained from the calibration curve by calculating the slope and mean standard deviation of the curve. The LOD and LOQ were found to be 0.21 µg/mL and 0.64 µg/mL, respectively, with suitable precision and accuracy.

### 2.3. Specificity

This method for docetaxel nanoparticles proved to fit its intended purpose with no elution of interfering peaks with either the mobile phase or the excipients of blank polymeric nanoparticles. The peak purity was determined for the standard and the sample with a purity angle less than the purity threshold, confirming no co-elution of interfering peaks. The purity angles for the standard and samples were found to be 1.187 and 1.620, which were less than the purity threshold of 1.211 and 1.906, respectively (Figure S1A–D).

### 2.4. Accuracy and Precision

The accuracy of the method was determined at three levels, i.e., 80%, 100%, and 120%. The levels of spiking each concentration in triplicate and the percentage recovery of spiked docetaxel was found to be 100.655%, 98.748%, and 100.604%, with RSD of 0.0568%, 0.3798%, and 0.0843%, respectively (Table 1).

The precision of the docetaxel nanoparticles was tested by determining the retention times of six replicates of a single sample. Their relative standard deviation was ascertained, which was ≤2%, per ICH guidelines (Table 1).

The intermediate precision was carried out on two different days. The results showed that the percentage assay of docetaxel was not significantly different from the RSD ≤ 2% similar to the peak areas, which were also within the limits of ≤2% RSD, depicting that the method is precise and reliable (Table 1).

### 2.5. Robustness

The robustness of the method was determined by intentionally altering the flow rate of the mobile phase (1.2 ± 0.2 mL/min), temperature (50 ± 5 °C), and wavelength (232 ± 2 nm). The obtained results depicted that these minor changes did not influence the robustness of the method, as the RSD obtained was within the defined criteria of ≤2%. Additionally, the tailing or asymmetry factor was always ≤2, and the average plate count, in any case, must not be less than 2000 per the CDER (Center for Drug Evaluation and Research) and USP (United States Pharmacopeia) requirements (Table S1).

### 2.6. System Suitability (SST)

System suitability is an integral part of every liquid chromatographic method, either developed or under development, to test the effect of analytical conditions on various parameters of the standard, such as retention time, mean peak area, tailing factor, and in the case of two components, resolution factor, etc. The system suitability parameters for this specific method are listed in Table S2, which meet the criteria of the allowable limits of SST per the USP/BP or other pharmacopeial guidelines.

### 2.7. Characterization of DTX-mPEG-PLA Nanoparticles

#### 2.7.1. Morphology, Particle Size, and Zeta Potential

**Figure 1:**
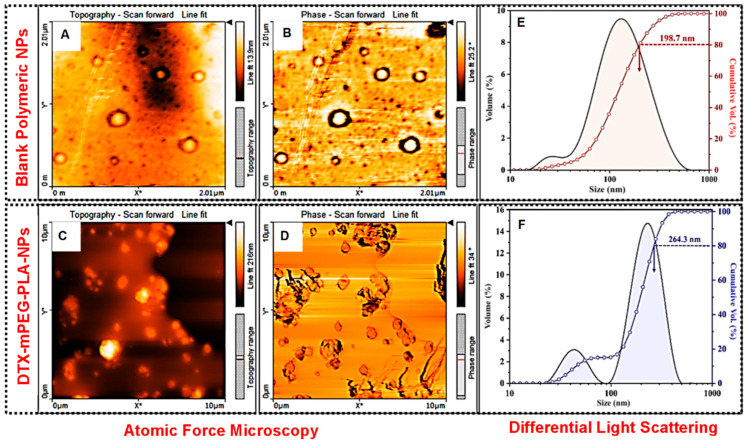
Particle size distribution and morphological analysis. (**A**) Topography image of B-NPs. (**B**) Differential phase micrographs of B-NPs. (**C**) Topography images of DTX-mPEG-PLA-NPs. (**D**) Differential phase micrographs of DTX-mPEG-PLA-NPs. (**E**) DLS of B-NPs, the cumulative particle size distribution curve, and D80 average particle size of B-PNs—198.7 nm. (**F**) DLS of DTX-mPEG-PLA-NPs, the cumulative particle size distribution curve, and D80 average particle size of DTX-mPEG-PLA-NPs—264.3 nm.

The surface morphology of the blank polymeric nanoparticles (B-NPs) and docetaxel-loaded nanoparticles (DTX-mPEG-PLA-NPs) was examined via atomic force microscopy (AFM), as shown in Figure 1B–E. The well-defined spherical shape B-NPs were formed during the DESE process, as validated in Figure 1B,C. Both the AFM test results and particle size distribution curves obtained from DLS analysis represented the formation of various size B-NPs. The cumulative volume % curve (Figure 1F) indicated that approximately 30 vol. % particles were <100 nm. However, a very small amount (<10 vol. %) of B-NPs were <40 nm in size, as indicated by the origin of a shoulder peak in the distribution curve. The average particle size (D80) of the B-NPs was found to be 198.7 nm (Table 2). On the other hand, DTX-mPEG-PLA-NPs exhibited angular shape particles with variable sizes, as shown in Figure 1D–F. The presence of docetaxel at the core of these particles can be confirmed from the phase differential scan, as shown in Figure 1F. The DLS analysis of the DTX-mPEG-PLA-NPs also showed two distinct peaks in the particle distribution curve, which corresponded to the formation of variable-sized particles. For instance, a well-defined shoulder peak represented the formation of ~10 vol. % of small-size (<50 nm) particles, as shown in Figure 1F. However, the average D80 particle size of the DTX-mPEG-PLA-NPs was found to be 264.3 nm (Figure 1F and Table 2). Compared to B-NPs, the increase in the size of the DTX-mPEG-PLA-NPs was attributed to the drug loading and encapsulation of the DTX particles within the mPEG-PLA.

Qualitatively, the negative zeta potential of the B-NPs (−16.47 ± 3.046 mV) represented their fairly good dispersibility in water. On the other hand, the relatively more negative (−33.79 ± 7.08 mV) zeta potential of the DTX-mPEG-PLA-NPs corresponded to their increased colloidal stability (Table 2). The quantitative information of the particle size analysis and zeta potential measurements is given in Table 2.

#### 2.7.2. Drug Incorporation Studies

Drug loading in polymeric nanoparticles is always a challenge. Previously, many water-insoluble drugs have been loaded to form nanoparticles, such as lidocaine [33] and prednisolone [34]. We attempted to improve the encapsulation efficiency of DTX, a hydrophobic drug using amphiphilic polymer, mPEG-PLA, which can self-assemble into micelles incorporating DTX in its core during the rehydration process. The obtained results demonstrated a drug loading of 1.47% and an encapsulation efficiency of 62.22%, which was higher than a previous report [34] (Table 2).

#### 2.7.3. In Vitro Drug Release Kinetics

**Figure 2:**
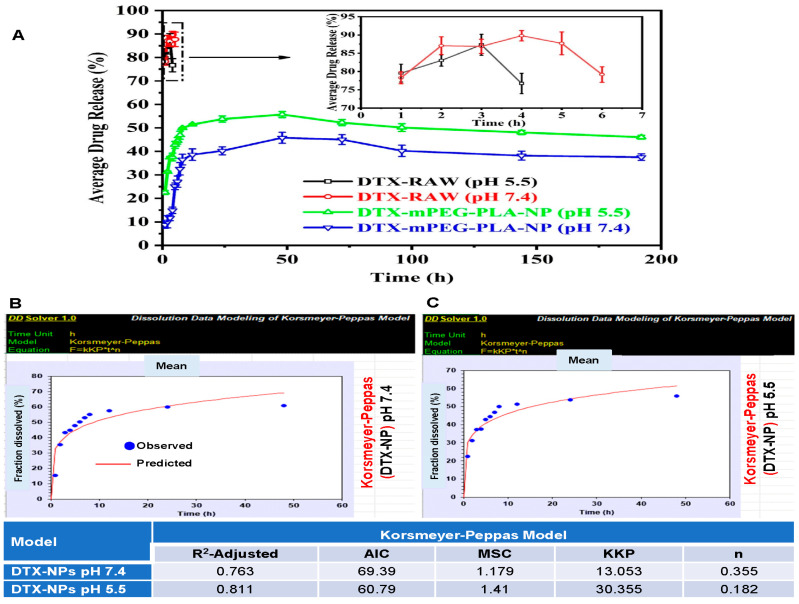
In vitro drug release kinetics. (**A**) In vitro drug release profiles of Free-DTX at pH 5.5 and 7.4 and DTX-mPEG-PLA-NPs. (**B**) Korsmeyer–Peppas mathematical model of drug release kinetics at pH 7.4. (**C**) Korsmeyer–Peppas mathematical model of drug release kinetics at pH 5.5.

The drug release from the polymeric nanoparticles depends on the biodegradability of the polymer, the molecular weight of the polymer, the stability of nanoparticles, and the pH of the medium. For instance, DTX-mPEG-PLA-NPs demonstrated an initial burst release within the first 8 h and continued to release the drug in a sustained manner up to a maximum of 45.12% and 55.71% at pH 7.4 and 5.5, respectively, as evident in Figure 2A,B. This slow release of docetaxel from nanoparticles at both pHs is indicative of a sustained release pattern. However, a slightly higher release at pH 5.5 is suggestive of a minor effect of pH on the behavior of nanoparticles. The release rate of Free-DTX was much higher compared to nanoparticles and exhibited approximately 90% and 87% of release in the first 4 h at pH 7.4 and 5.5, respectively (Figure 2A,B).

The data obtained via the Korsmyere–Peppas mathematical model, most commonly used to interpret the non-linear diffusion profiles, demonstrated a Fickian diffusion with an ‘n’ value of ≤0.45 at both pHs, suggesting anomalous transport, i.e., diffusion and swelling (Figure 2B,C).

### 2.8. MCF-7 Breast Cancer Cells form Mammospheres in Anchorage-Independent Culture Conditions

**Figure 3:**
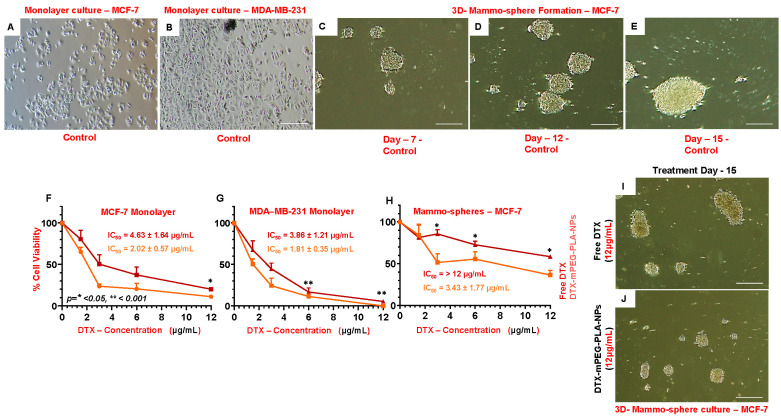
Cell viability MTT assay. (**A**) Micrograph of MCF-7 2-D monolayer culture. (**B**) Micrograph of MDA-MB-231 monolayer culture. (**C**–**E**) Mammosphere generation, micrograph of 3D culture from day *7* to day 15. (**F**) Percent cell viability of MCF-7 cells *against* DTX-mPEG-PLA-NPs and Free-DTX in 2D culture. (**G**) Percent cell viability of MDA-MB-231 cells against DTX-mPEG-PLA-NPs and Free-DTX in 2D culture. (**H**) Percent cell viability of MCF-7 cells against DTX-mPEG-PLA-NPs and Free-DTX in 3D mammosphere culture. (**I**,**J**) Micrographs of MCF-7 3-D mammospheres after 15 days of incubation with Free-DTX (**I**) and DTX-MPEG-PLA-NPs (**J**). IC50 half maximum inhibitory concentration, (Scale bar; (**A**–**E**) and (**I**,**J**): 200 μm). Results were represented as mean ± SD (n = 3). Asterisk (*) represents the significant alpha values.

The mammospheres were generated employing MCF-7 cells to enrich cancer stem-like cells. The development of mammospheres under the conditions mentioned in the method section was observed for 15 days. As shown in Figure 3B–E, the single-cell suspension seeded on day 0 started to form small clumps, which became small suspended colonies on day 3 (Figure 3B). From day 5 (Figure 3C) onward the size of the mammospheres started to increase (Figure 3B–E)—with a mammosphere formation efficiency (MFE) of 8.47% ± 3.58 on day 15.

### 2.9. Effect of DTX-mPEG-PLA-NPs on Cell Viability and Clonogenicity

MTT assay was performed using two different cultures, i.e., two-dimensional (2D, mono-layer) MCF-7 and MBA-MD-231, and three-dimensional (mammospheres) MCF-7 culture system only. In the 2D mono-layer culture, compared to Free-DTX, DTX-mPEG-PLA-NPs showed significant cytotoxicity against MCF-7 (Figure 3F), and with the same concentrations, significant and even better cytotoxic effects were observed against MDA-MB-231 cells (Figure 3G). However, in the 3D MCF-7 culture (mammospheres), the cytotoxic effects, in terms of % viability, were significantly higher at all three concentrations (Figure 3H), as evidenced by the mammosphere size at day 15 in DTX-mPEG-PLA-NPs compared to Free-DTX (Figure 3I,J).

The differences in the sensitivity of DTX-NPs to MDA-MB-231 and MCF-7 cell lines were obvious from IC_50_ values, measured with Graph Pad Prism 5 software (San Diego, CA, USA). For MDA-MB-231 cells, the IC_50_ values were 3.86 ± 1.21 μg/mL and 1.81 ± 0.31 μg/mL for Free-DTX and DTX-mPEG-PLA-NPs, respectively. For MCF-7 cells, the IC_50_ values were 4.63 ± 1.64 μg/mL and 2.02 ± 0.57 μg/mL for Free-DTX and DTX-mPEG-PLA-NPs, respectively. For MCF-7 mammospheres, the difference was enormous, the IC_50_ values were >12 μg/mL and 3.43 ± 1.77 μg/mL for Free-DTX and DTX-mPEG-PLA-NPs, respectively.

**Figure 4:**
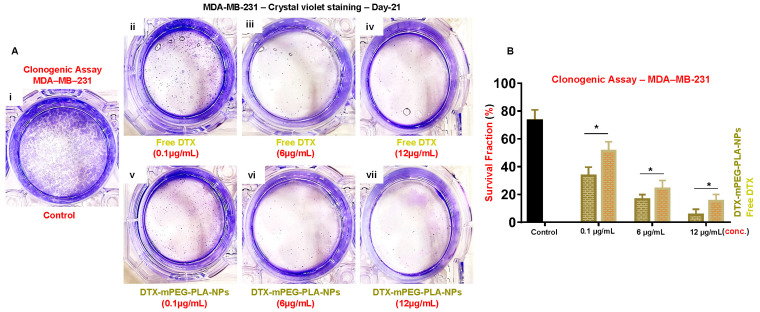
Clonogenic assay. (**Ai**–**vi**) Crystal violet staining of MDA-MB-231 cells after 21 days of incubation with DTX-mPEG-PLA-NPs and Free-DTX in 2D culture. (**Aii**–**iv**) Inhibition of the clonogenic potential of MDA-MB-231 cells at day 21 against 0.1, 6, and 12 μg/mL concentrations of Free-DTX. (**Av**–**vii**) Inhibition of the clonogenic potential of MDA-MB-231 cells at day 21 against 0.1, 6, and 12 μg/mL concentrations of DTX-MPEG-PLA-NPs. (**B**) Survival fraction of MDA-MB-231 cells after treatment with 0.1, 6, and 12 μg/mL concentrations of DTX-mPEG-PLA-NPs and Free-DTX. A represents the figure panel, while Roman number (i or ii) represents the number of figures in panel A. Asterisk (*) represents the significant alpha values.

The clonogenic assay tests the capacity of every cell in a population to undergo “unlimited” division and is used to assess the long-term efficacy of cytotoxic agents [35]. As shown in Figure 4A,B, compared to Free-DTX (Figure 4Aii–iv,B), DTX-mPEG-PLA-NPs demonstrated significant inhibitions of the clonogenic potential of MDA-MB-231 for all concentrations (Figure 4Av-vii,B). The survival fraction (SF) significantly reduced after treatment with DTX-mPEG-PLA-NPs, i.e., below 7% in 12 μg/mL conc., taking into account the plating efficiency of 73.4% in the control (Figure 4B).

## 3. Discussion

Several previous reports claim to develop a rapid, sensitive, and reliable HPLC method for the detection of docetaxel (DTX) in biological matrices [36,37]. Attempts have also been made to develop and validate methods to detect DTX-loaded PLGA nanoparticles [38]. More recently, using the amphiphilic polymer, mPEG-PLA, DTX was loaded on this polymer for sarcoma therapy [4]. However, method validation for DTX loaded onto mPEG-PLA has not been reported yet. This study, for the first time, reported a rapid, sensitive, and robust HPLC method for the detection of DTX in mPEG-PLA. Data suggested that the developed method showed a linear response in the acceptable range, suitable specificity, accuracy/precision, and robustness per ICH guidelines. Moreover, DLS analysis showed variable particle sizes of DTX-mPEG-PLA-NPs with an average particle size of 264.3 nm greater than the blank NPs. This change in the size of DTX-mPEG-PLA-NPs compared to blank NPs can be attributed to drug encapsulation and the properties of the polymer matrix. [39,40]. While the negative zeta potential corresponds to colloidal stability. The increased colloidal stability is primarily due to steric stabilization provided by the mPEG-PLA polymer, preventing particle aggregation and enhancing dispersion. In other words, DTX (docetaxel) tends to aggregate in aqueous solutions due to its hydrophobic nature. When encapsulated within the NPs, the drug is shielded from the surrounding environment, reducing the likelihood of drug aggregation and precipitation. These factors are important for the successful delivery of drugs in nanoparticle-based formulations [41,42]. Drug release was bi-phasic as evident in the Korsmyere–Peppas model—a Fikcian diffusion suggestive of anomalous transport, i.e., diffusion and swelling. Compared to Free-DTX, DTX-mPEG-PLA-NPs demonstrated significantly higher cytotoxic potential in 2D and 3D MCF-7 cell cultures. Similar results were obtained using MDA-MB-231 monolayer culture. Moreover, DTX-mPLEG-PLA-NPs also showed significant anti-clonogenic potential against the MDA-MB-231 cell line.

The quantitative determination of analytes and drug metabolites in the biological matrix plays an important role in the estimation and interpretation of bioavailability, bioequivalence, and pharmacological and pharmacokinetic profiles of a drug [36]. Data suggested that the specificity, accuracy, and precision followed ICH guidelines. The linearity of any analytical procedure, in a given range, determines the ability of a procedure to obtain results that are directly proportional to the concentration of the sample. The validated method was found to exhibit a linear response in the range of 0.64–120 µg/mL (r^2^ = 0.997), corroborating a previous report where DTX was simultaneously estimated with ritonavir in PLGA nanoparticles [38]. Additionally, inter-day precision and repeatability, the degree of agreement among individual tests, accuracy, and the closeness of the results to the true value, of DTX-mPEG-PLA-NPs exhibited RSD values of ≤2%., i.e., no significant difference in the assay when tested within and between days, confirming previous report [37]. The robustness of any analytical method plays an important role during method development [43]. The robustness of the system was checked after deliberate alterations in the various parameters, i.e., flow rate, temperature, and wavelength, yet changes in the operational parameters did not show any significant changes in the performance of the system—asymmetry factor below 2, the number of theoretical plates ≥2000, and peak area RSD ≤ 2%—complying with the Centre for Drug Evaluation and Research (CDER-FDA) and USP guidelines [44].

The synthesis and characterization of DTX-mPEG-PLA-NPs for sarcoma therapy have been described recently [4]. Compared to the reported particle size of 100–123 nm and PDI of 0.078–0.238, we observed an average particle size of 264.3 nm and PDI of 0.524. The probable reason for these differences could be the organic phase, the used emulsifier, and lyophilization protectants. Nevertheless, despite the complexity of the prepared NPs, the PDI of the nanoparticles was below 0.7, complying with the International Standards Organization (ISO)—mostly attributed to a more heterogenous or broad size [45]. The zeta potential is the qualitative measure of charge on the particle surface and is highly dependent on the pH, temperature, and concentration of the phases present in the dispersant. A higher value of zeta potential approaching approximately ±30 mV represents a high physicochemical stability of the dispersion. This highlights the existence of electrostatic repulsive forces between particles that could avoid aggregation of the NPs [46]. However, without knowing the Van der Waal attractive forces, it is difficult to predict the quantitative value of the surface charge density of the NPs.

The possible mechanisms of drug release from nanoparticles could be either due to the desorption of the drug from the nanoparticle surface or erosion of polymeric nanocarrier, i.e., the diffusion of the drug from the matrix or diffusion from the wall of nanoparticles, or a combination of both, i.e., diffusion and erosion [47]. Regardless of the pH, the release rate of Free-DTX was up to 80% in the first few hours, while loading onto the polymer resulted in a release rate of 45–56%—demonstrating that DTX-mPEG-PLA-NPs were able to sustain the release of DTX at a bloodstream pH of 7.4 and a pH of tumor extracellular environment ~5.5 [48]. The burst release phenomenon of Free-DTX was very obvious within 8 h. While only 25–30% remained in the membrane, which after 12 h reached the outer phase. The greater retention of Free-DTX could be attributable to membrane quality and saturation point in the sink. The applied Korsmeyer–Peppas model [49], usually employed to describe drug release from the polymer matrix, with high R^2^-adjusted values and ‘n´ value of ≤ 0.45 at both pHs, suggested anomalous transport, i.e., diffusion and swelling. The in vitro and in vivo cytotoxic potential of DTX-NPs in combination with bortezomib [5], and DTX loaded onto mPEG-PLA [4], respectively, have been demonstrated before. In a monolayer 2D culture of MCF-7, compared to Free-DTX, DTX-mPEG-PLA-NPs demonstrated significant cytotoxicity at a dose of 12 ug/mL, while these differences appeared more significant in the mammosphere 3D culture, a way to enrich cancer-like stem cells employing anchorage-independent culture conditions [50], a pre-cancerous model, and a way of enriching cancer stem cells, where epithelial cells tend to survive better and play important role in establishing barrier tissue—crucial for the survival of cells in many diseases [51]. The cytotoxic effects were more pronounced against MDA-MB-231 cells at all concentrations—corroborating previous findings that MCF-7 exhibited greater resistance against docetaxel in comparison to MDA-MB-231 cells [52]. Cancer cells have the unique potential of self-renewal, supported and coached by cancer stem cells; thus, a single cancer cell can form colonies, thus, the clonogenic method was opted that has widely been used to determine cell reproductive death after treatment with cytotoxic agents [53,54]. Similarly, we observed that DTX-mPEG-PLA-NPs, compared to Free-DTX, showed significant inhibition of the clonogenic potential of MBA-MD-231 cells in all concentrations.

Taking originality into account, this study not only establishes a robust analytical method for DTX quantification but also introduces DTX-mPEG-PLA-NPs as a promising, biocompatible drug delivery system with remarkable cytotoxic and anti-clonogenic potential. These findings hold great promise in the realm of breast cancer therapeutics, offering the potential for improved treatment outcomes. Beyond the methodological advancements, we have explored the dynamic drug release kinetics of DTX-mPEG-PLA-NPs, revealing a complex pattern that conforms to the Krosmeyers–Peppas model. This model uncovers initial biphasic release kinetics followed by a multistep sustained release over an extended duration of 190 h. Moreover, the developed nanoparticles have the potential to enhance the blood circulating capacity of the drug by preventing the attacks by macrophages, while at the same time, nano-size reduces uptake by the kidney or liver—a critical determinant of drug efficacy [13]

## 4. Materials and Methods

Docetaxel was provided by IMA S.A.I.C Laboratories Argentina. Poly(l-lactide)2k- Methoxyl poly (ethylene glycol)2k was purchased from Nanosoft Biotechnology LLC, NC, USA. Ethyl acetate was purchased from Penta Chemicals Unlimited under the trade name of Ing. Petr Svec-Penta s.r.o. Polyvinyl alcohol was purchased from Sigma-Aldrich, Darmstadt, Germany. Baker Analyzed HPLC grade Acetonitrile was purchased from Avantor Performance Materials Korea Limited. All other reagents were of analytical grade unless otherwise indicated.

### 4.1. Preparation of DTX-mPEG-PLA Nanoparticles

The double emulsion solvent evaporation technique was adopted for nanoparticle preparation with some modifications in the reported method [55]. The aqueous phase was dropwise added in a continuous vortexing organic phase (Ethyl acetate) comprising polymer (10 mg/mL) and docetaxel (0.1 mg/mL) [56] with polysorbate 80 (25 µL/mg docetaxel). This water-in-oil emulsion was probe sonicated (Soniprep 150) for one minute while cooling in an ice bath. Polyvinyl alcohol 1.0% solution was added dropwise with continuous vortexing for a further 30 s. The aqueous-to-organic phase ratio was set to 3:1 and was probe sonicated for another 3 min before cooled on the ice bath. This resultant w/o/w emulsion was magnetically stirred for 2.5 h to evaporate the organic contents, followed by centrifugation at 14,000 rpm for 30 min, which were then washed twice with water to remove any remnant of poly (vinyl alcohol) (PVA), followed by lyophilization.

Drug loading and encapsulation efficiency were calculated via the HPLC system using Spherisorb ODS2 column (4.6 mm × 150 mm × 5 µm) of Waters (Milford, MA, USA) at 50 °C temperature. The wavelength for detection was selected to be 232 nm with an injection volume of 40 µL. The flow rate was set to 1.2 mL/min. Both DL and EE were estimated using the following formulas:

$$\mathrm{Drug}\;\mathrm{Loading}\;(\mathrm{DL},\;W/W\;\%)=\frac{\mathrm{Weight}\;\mathrm{of}\;\mathrm{drug}\;\mathrm{in}\;\mathrm{Nanoparticles}}{\mathrm{Weight}\;\mathrm{of}\;\mathrm{total}\;\mathrm{in}\;\mathrm{Nanoparticles}}\times 100$$

$$\mathrm{Encapsulation}\;\mathrm{Efficiency}\;(\mathrm{EE},\;W/W\;\%)=\frac{Weight\;of\;drug\;in\;nanoparticles}{\mathrm{Weight}\;\mathrm{of}\;\mathrm{total}\;\mathrm{drug}\;\mathrm{used}\;\mathrm{in}\;\mathrm{formulation}}\times 100$$

### 4.2. Characterization of Polymeric Nanoparticles

#### 4.2.1. Morphology

Atomic force microscopy (AFM) has been widely employed to study the 3D morphology of nanoparticles [57]. Therefore, the topography of the polymeric and docetaxel-loaded polymeric nanoparticles was observed via Nanosurf FlexAFM. Tap190AI g cantilever with Tapping mode was employed. Samples were prepared on a MICA sheet substrate by applying suspended particles with the help of capillary tubes.

#### 4.2.2. Particle Size

Malvern NanoZetasizer was used for particle size estimation, which followed the principle of dynamic light scattering (DLS) [58]. The particle size and size distribution were measured at 25 °C with a measurement position of 5.50 mm. The samples of nanoparticles were diluted in deionized water appropriately before measuring particle size.

#### 4.2.3. Zeta Potential

Zeta potential was measured using Zeta-Meter 3.0+ (ZM3-U g Somatco, Miami Beach, FL, USA). Briefly, the nanoparticles in water were dissolved and placed in the electrophoresis cell, and the electric field was activated. The values for zeta potential were recorded as the average of four measurements.

#### 4.2.4. In Vitro Drug Release Kinetics

The free DTX and DTX-loaded nanoparticles were suspended in 3 mL phosphate buffer (pH 7.4 and pH 5.5) in dialysis bags. The dialysis bags were hung in a 50 mL tube containing external media, and the whole system was incubated at 37 °C on a shaker mixer at 100 revolutions per minute. At predetermined time intervals, 3 mL of the release media was withdrawn and refilled with fresh incubation media. The amount of drug release was quantified using a UV-visible spectrophotometer (Shimadzu UV-1800 Double Beam) as published in the literature [59] at λ_max_ 231. The experiments were conducted in triplicates.

For drug release kinetics, the first 60% of data were fitted in the Korsmeyer–Peppas model and analyzed using the DDSolver software, version 1.0 [60]. The R^2^-adjusted values were found to be high in the Korsmeyer–Peppas model usually employed to describe drug release from the polymer matrix. The value of *n* ≤ 0.45 at both pHs characterizes the release mechanism of a drug and corresponds to a Fickian diffusion mechanism, which is applicable when a drug has to move through the polymeric matrix into the release medium, and the polymer chains are the diffusional barriers restricting the drug release. The parameters were calculated following the step-by-step utilization of equations employing an Excel spreadsheet. The *R* adjusted ^2^ value was used as the model selection criterion with the best model *R*^2^ value closest to 1.

### 4.3. Instrumentation

A Waters AQUITY Arc (Serial NO. A18VQS616G) HPLC system (Milford, MA USA) was used for method development. The system was equipped with photodiode array (PDA) detector (Waters 2998), column compartment with temperature control, Sample Manager FTN-R with autosampler, Quaternary Solvent Manager-R with online degasser, and quaternary pump. The data acquisition and reporting were performed employing Empower software of chromatography, version 2 (Milford, MA, USA).

### 4.4. Preparation of Standard and Sample Solutions

A diluent consisting of acetonitrile and water was prepared in a 1:1 ratio. DTX standard was first dissolved in 5% ethanol and then in the diluent (acetonitrile). A concentration of 10 µg/mL of standard DTX was prepared. The samples of nanoparticles were also dissolved and diluted with the same diluent. Both, the standard and the sample were filtered through a 0.22 µm filter (Millipore, Bedford, MA, USA) before analysis.

### 4.5. Method Validation

Details on the various parameters, such as accuracy, precision, linearity, specificity, and robustness are given in Supplementary Materials.

### 4.6. Mammosphere Formation Assay

#### 4.6.1. Generation of Mammospheres from MCF-7 Cells

To determine anchorage-independent growth, mammospheres were generated employing MCF-7 cells as described previously [61]. Briefly, 80% confluent MCF-7 cells were trypsinized and transferred to a 15 mL falcon tube for centrifugation (Micro 200, Hettich Zentrifugen). The pellet obtained was resuspended in freshly prepared mammosphere media (DMEM/F12 with L-glutamine, Capricorn Scientific GmbH, Ebsdorfergrund, Germany), 100 U/mL Penicillin, 100 U/mL Streptomycin, 20 ng/mL recombinant hEGF (ABclonal, Woburn, MA, USA), and 10 ng/mL recombinant hbFGF (ABclonal)). The suspension was passed via a 25 G needle thrice to ensure single-cell suspension. After counting, the viable cells were seeded in a T-25 ultra-low attachment flask (Corning Incorporated, Corning, NY, USA) at a density of 1000 cells/cm^2^ [62] and incubated at 37 °C, 5% CO_2,_ and 95% relative humidity for 15 days. The flask remained un-disturbed, especially for the first 5 days. The images were taken on days 0, 3, 7, 12, and 15 under a bright field microscope. Thereafter, mammospheres formed with a diameter greater than 40 µm were counted using a microscope fitted with a graticule, and mammosphere formation efficiency (MFE) was calculated using the formula:

$$\left(MEF=No.\;of\;mammospheres\;formed\;per\;well÷No.\;of\;cells\;seeded\;per\;well\times 100\right)$$

#### 4.6.2. Treatment of Mammospheres

The effects of DTX-mPEG-PLA-NPs and Free-DTX drug were characterized by treating the mammosphere with various drug concentrations. After lifting the cells, the cells were seeded in triplicates at a density of 1000 cells/well in a 96-well ultra-low attachment plate (Corning^®^ Costar^®^ 7007) in mammosphere media, followed by incubation for 24 h. Thereafter, per the literature evidence, the cells were exposed to various concentrations of DTX-mPEG-PLA-NPs and Free-DTX in three different concentrations [63], i.e., 0.1 µg/mL, 6.0 µg/mL, 12.0 µg/mL for 15 days.

### 4.7. MTT Assay

#### 4.7.1. Two-dimensional Culture

Briefly, both MCF-7 and MBA-MD-231 cells, 3.7 × 10^4^ cells/well, were seeded into 96-well plates using (DMEM/F12 with L-glutamine, Capricorn Scientific GmbH), 10% Fetal Bovine Serum (Sigma-Aldrich) and 1% 100 U Penicillin/100 U Streptomycin (Caisson Laboratories, Inc., Smithfield, UT, USA), incubated at 95% relative humidity, 5% CO_2_ and 37 °C for 24 h to allow attachment of cells. Then, cells were treated in triplicates with DTX-mPEG-PLA-NPs and Free-DTX drug in three different concentrations, i.e., 0.1 µg/mL, 6.0 µg/mL, 12.0 µg/mL [63], followed by incubation for 24 h. Thereafter, 10 µL (5 mg/mL) MTT Solution (Bio World) was added to each well, followed by incubation for 3 h. Finally, the media was aspirated, and the formed crystals of formazan were solubilized by adding 100 µL dimethyl sulfoxide (DMSO), followed by continuous shaking of the plate for 30 min. The optical densities were obtained at 570 nm using a microplate reader. The wells with only cells and culture medium without any treatment were considered controls.

#### 4.7.2. Three-dimensional Culture

For cell viability, an MTT assay was conducted on DTX-mPEG-PLA-NPs and Free-DTX drug-treated mammospheres (*n* = 3) on day 15. Briefly, 10 µL (10 mg/mL) of MTT solution (Bio World) was added to each well, followed by incubation for 5 h. The rest of the procedure was the same as described above for the 2D culture, except for the continuous shaking with DMSO for an hour.

### 4.8. Clonogenic Assay

The clonogenic assay is a versatile in vitro screening tool that assesses each cell’s potential for “unlimited” division and is the preferred method for evaluating the efficacy of cytotoxic agents [35].

The assay was carried out as described previously [35]. MDA-MB-231 cells were seeded at a density of 2 × 10^6^ cells per well in 6-well plates and incubated at 37 °C for 24 h. After incubation, cells were treated with 2 mL of DTX-mPEG-PLA-NPs and Free-DTX at concentrations of 0.1, 6, and 12 µg/mL for 24 h, while control wells received 2 mL of fresh media. The cells were then harvested and counted, and 500 cells were re-plated in 6-well plates to assess their ability to form colonies. After 21 days of re-plating, colonies, consisting of at least 50 cells or more were fixed and stained with crystal violet dye. The colonies were counted using a microscope, and the percentage of colony formation relative to the control was calculated.

The surviving fraction (SF) was determined by dividing the number of colonies formed in treated cells by the number of cells seeded and multiplying the result by 100%. Plating efficiency (PE) was calculated by dividing the number of colonies formed in untreated cells by the number of cells seeded and multiplying the result by 100%.

## 5. Conclusions

Taken together, this is the first report on the development of a simple, accurate, and reproducible validation method of DTX in DTX-mPEG-PLA-NPs. The synthesized DTX-mPEG-PLA-NPs had an average size of 264.3 nm with a net negative charge and encapsulation efficiency of 62.22%. The in vitro drug release kinetic was explained by the Korsmeyer–Peppas model demonstrating biphasic response, with an initial release followed by a multistep slow and sustained release for more than 190 h following Fickian diffusion. With optimal encapsulation rate, uniform particle size, and negative zeta potential, the developed DTX-mPEG-PLA-NPs exhibited in vitro cytotoxicity against both MCF-7 and MDA-MB-231 breast cancer cell lines in 2D and MCF-7 in 3D culture (mammospheres) systems. Likewise, compared to free DTX, DTX-mPEG-PLA-NPs significantly inhibited the clonogenic potential of MDA-MB-231 cells.

## Figures and Tables

**Table 1:** Validation parameters of docetaxel nanoparticles (DTX-mPEG-PLA-NPs).

Accuracy of DTX-mPEG-PLA-NPs				
Level of Spiking	Recovery (*%*)	Mean Recovery (*%*)	Relative Standard Deviation (RSD, %)	
80%	100.663	100.655	0.0568	
	100.707			
	100.594			
100%	99.132	98.748	0.3798	
	98.729			
	99.382			
120%	100.526	100.604	0.0843	
	100.694			
	100.592			
**Repeatability and Inter-day Precision of DTX-mPEG-PLA-NPs**				
**Sample Name**	**Peak area—*Day 1***	**Docetaxel Recovery (*%*)—*Day 1***	**Peak area—*Day 2***	**Docetaxel Recovery (*%*)—*Day 2***
Replicate 1	20,462	99.141	20,429	98.981
Replicate 2	20,451	99.087	20,440.5	99.036
Replicate 3	20,461.5	99.138	20,448.5	99.075
Replicate 4	20,436.5	99.017	20,458.5	99.124
Replicate 5	20,411	98.893	20,452.5	99.095
Replicate 6	20,447	99.068	20,434.5	99.008
Mean	20,444.83	99.057	20,443.917	99.053
Standard Deviation	19.127	0.0846	11.227	0.0544
RSD	0.0936	0.0854	0.0549	0.0549

**Table 2:** Physical characterization of the docetaxel nanoparticles (DTX-mPEG-PLA-NPs).

Samples (*n* = 3)	Particle Size (*Average, nm*)	Polydispersity Index (*PDI*)	Zeta Potential (mV) (*Avg ± SD*)	Encapsulation Efficiency (*EE%*)
Blank NPs	198.7 ± 7.71	0.298	−16.47 ± 3.046	-
DTX-MPEG-PLA-NPs	264.3 ± 7.28	0.524	−33.793 ± 7.08	62.22 ± 1.45

## Data Availability

All raw data used in this publication are available from the corresponding author(s) upon reasonable request.

## Supplementary Materials

The following supporting information can be downloaded at: https://www.mdpi.com/article/10.3390/ph16111600/s1, Figure S1. Linearity calibration curve for the docetaxel drug concentration (limit of detection and limit of quantification). Figure S2: HPLC chromatograms. (A) Mobile phase. (B) docetaxel standard. (C) Blank polymeric nanoparticles. (D) DTX-mPEG-PLA nanoparticles. Table S1. Robustness of the validation method for docetaxel nanoparticles (DTX-mPEG-PLA-NPs). Table S2. System suitability parameters (per USP and CDER guidelines).
