# Supplementary material for: Docetaxel-Loaded Methoxy poly(ethylene glycol)-poly (L-lactic Acid) Nanoparticles for Breast Cancer: Synthesis, Characterization, Method Validation, and Cytotoxicity
Source: Pharmaceuticals (Basel). 2023 Nov 13;16(11):1600. doi: 10.3390/ph16111600 (PMC10675362; doi:10.3390/ph16111600)
Supplement: Supplementary file 1 [file pharmaceuticals-16-01600-s001.zip › pharmaceuticals-2654500-supplementary.pdf]

Method Validation

The method validation was carried out following the International Conference on Harmonization (ICH) guidelines to test the suitability of the analytical procedure for its intended use and further study multiple variates to check the legitimate reliability and usefulness of the analytical procedure.

Accuracy: the accuracy of the method was established by calculating the percentage recovery of the mean concentration of DTX spiked at three different concentrations i.e., 80%, 100%, and 120% along with the calculation of relative standard deviation (RSD). The mean concentration found at each level was compared to the theoretical value which was considered 100%.

Precision: the precision of the DTX-NPs was determined at the levels of repeatability and intermediate precision. The repeatability of the sample was assessed by measuring six replicates of 100 % test concentration and the results were reported as relative standard deviation (RSD). The intermediate precision was carried out by analyzing six replicates of 100 % concentration on two different days and the precision results were expressed as RSD.

Linearity: The linearity of the method was determined by drawing a regression line when a plot is drawn between the mean peak area and the varying concentrations of the standard solution, by employing the linear regression least square method. The Limit of Detection and Limit of Quantification was determined from the calibration curve drawn with linearity data with the formulas: $LOD = Standard\ deviation/Slope \times 3.3$

$$LOQ = Standard\ deviation/Slope \times 10$$

Specificity: The specificity of the method was determined by comparing the chromatograms of blank polymeric nanoparticles and drug-loaded polymeric nanoparticles along with the standard chromatogram. For a method to be specific, there should be no interference of the mobile phase or excipients on the spectrum of the moiety of interest during analysis.

Robustness: robustness of the method was evaluated by varying the wavelength ±2 nm, analytical column temperature to ± 5 °C, and flow rate between ±0.2ml/min

Supplementary Information

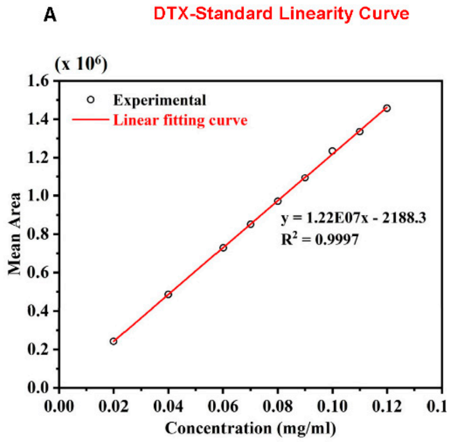

Figure S1A. Linearity calibration curve for the Docetaxel drug concentration (limit of detection and limit of quantification).

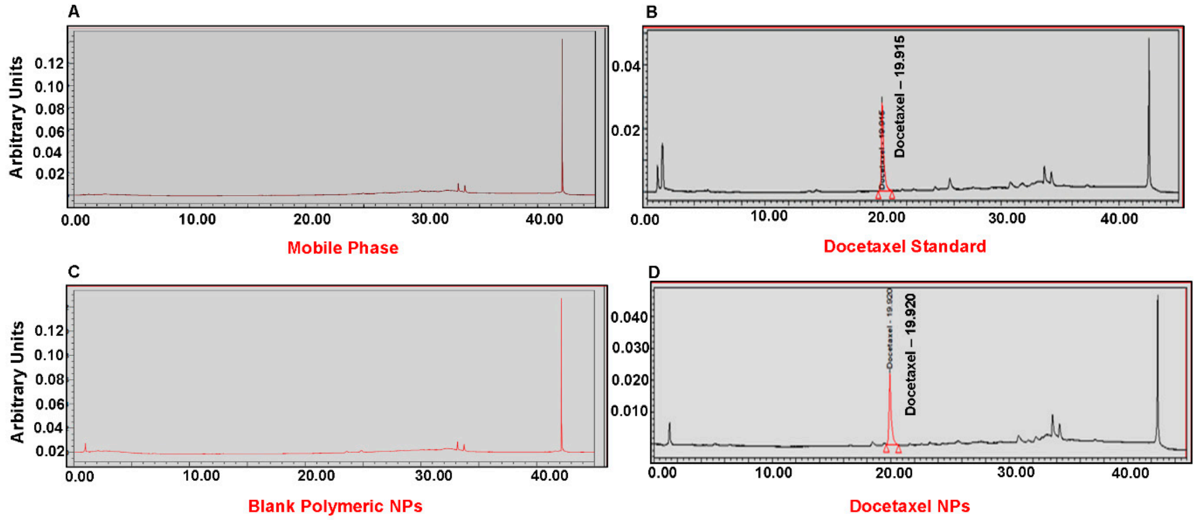

Figure S2: **HPLC chromatograms. (A)** Mobile phase. **(B)** Docetaxel standard (free). **(C)** Blank polymeric nanoparticles. **(D)** DTX-mPEG-PLA nanoparticles.

Table S1: *Robustness of the validation method*

**Changes in Parameters**	**Mean % recovery ± RSD**	**Mean Retention Time ± RSD**	**Asymmetry factor**	**Average Plate Count**
**None**	99.057 ± 0.085	19.28 ± 0.167	1.12	122140.33
**Wavelength 230 nm**	98.503 ± 0.138	19.803 ± 0.029	1.18	58199.67
**Wavelength 234 nm**	97.836 ± 0.226	19.803 ± 0.029	1.22	63141.67

**Temperature 45 °C**	99.786 ± 0.269	19.75 ±0.0506	1.80	25569.33
**Temperature 55 °C**	96.867 ± 0.446	19.07 ± 0.0908	1.01	53890.67
**Flow rate 1.0 ml/min**	97.052 ± 0.285	20.886 ± 0.0276	1.25	48950.33
**Flow rate 1.4 ml/min**	97.446 ± 0.142	19.07 ± 0.0908	1.05	53591.67

Table S2. *System suitability parameters*

Parameters	Mean ± RSD (%)
**Retention time**	19.91 ± 0.038
**Peak area**	290033.2 ± 0.387
**Peak Tailing**	1.82 ± 1.54
